# Establishment success of sooty beech scale insects, Ultracoelostoma sp., on different host tree species in New Zealand

**DOI:** 10.1673/2006_06_29.1

**Published:** 2006-10-20

**Authors:** Carl W. Wardhaugh, Raphael K. Didham

**Affiliations:** School of Biological Sciences, University of Canterbury, Private Bag 4800, Christchurch, New Zealand

**Keywords:** honeydew, host preference, local adaptation, Ultracoelostoma assimile, Ultracoelostoma brittini, Nothofagus fusca, Nothofagus solandri

## Abstract

The sooty beech scale insect (Ultracoelostoma sp.) (Hemiptera: Margarodidae) exhibits a highly patchy distribution at local and regional scales. A major factor driving this common distributional phenomenon in other phloem-feeding insects is aggregation and local adaptation. The aim of this study was to determine if Ultracoelostoma was locally adapted to its natal host trees, by contrasting the establishment rates of first instar “crawlers” in reciprocal transfers to natal versus novel hosts. Although there are two closely-related species of sooty beech scale insect, the morphological characters of crawlers in this study were intermediate between those of U. assimile and U. brittini. However, all of the voucher specimens examined had consistent morphology, indicating that they belong to one species which we refer to as Ultracoelostoma sp. Reciprocal transfers of crawlers were carried out between individual red beech (Nothofagus fusca), as well as between mountain beech (N. solandri) and red beech trees, to ascertain if insects had become locally adapted to their individual host tree or to host species. In total, 480 crawlers were placed in enclosures on their natal and novel host trees, of which only 32 (6.7 %) became established. No evidence for local adaptation, either to individual host trees or to host tree species, was found. There was also no difference in crawler establishment between natal and novel hosts. However, crawlers originating from mountain beech trees had significantly higher establishment rates on both natal mountain beech and novel red beech hosts, than did crawlers originating from red beech trees. The superior ability of mountain beech crawlers to become established, even on novel red beech trees, suggests that scale insects on mountain beech trees have higher individual fitness (possibly due to maternal effects mediated by differences in host nutritional quality, defensive compounds or growth rate). This increased fitness may result in crawlers being better provisioned to search for appropriate establishment sites. The results of this study indicate that beech scale insects perform better on mountain beech at this site, although crawlers did not preferentially establish on mountain beech.

## Introduction

Herbivorous insects are not randomly dispersed among their host plants (Downing 1986), but rather they attack some species or individuals preferentially over others. This could be the result of several factors, such as the dispersal abilities of the insect, environmental variation, the heterogeneous effects of parasites and predators, or variation between host plants in their nutritional quality, susceptibility or defensive phenotypes (Strauss 1990). Local adaptation to these factors has been found in many polyphagous insect species, with genetically distinct populations forming on different host species (Mopper et al. 1984; Moran 1984; Via 1984; Pashley 1986, 1988; Feder et al. 1988; Roininen et al. 1993; Downie and Granett 1999; but see Faeth et al. 1981). For example, Via (1984) found that the pea aphid, Acyrthosiphon pisum, had significantly higher performance on its natal species (alfalfa for some individuals, and clover for other individuals), despite both host plants being in proximity to one another. Alternatively, some host species or individuals may simply be more susceptible to herbivorous insects (Fox and Morrow 1981; Mopper et al. 1990, 1991; Price 1991; Preszler and Price 1995). Thus, the heterogeneous distribution of some insect species on their host plants could be a function of the varying degrees of susceptibility in the host plant population. For example, Mopper et al. 1990 found that the survival rate of the sawfly, Neodiprion edulicolis, was significantly higher on susceptible pinyon pines, Pinus edulis, compared to resistant trees.

Many species of insects produce hundreds of generations on an individual host tree (Mopper et al. 1995). Phenotypic variation in long-lived host plants presents herbivorous insects with a patchy resource that they may become adapted to if the right conditions prevail (Van Zandt and Mopper 1998). Thus, the distribution of heterogeneous plant defensive phenotypes can lead to the evolution of herbivorous insects into locally adapted subpopulations, or demes, on individual trees within single host species (Cobb and Whitham 1993). Many species of herbivorous insects have been found to form genetically distinct demes on individual host plants since the hypothesis was first proposed by Edmunds and Alstad (1978) (e.g., Alstad et al. 1980; Wainhouse and Howell 1983; Alstad and Edmunds 1987; Karban 1989; Komatsu and Akimoto 1995; Mopper et al. 2000). For example, Mopper et al. (2000) showed that demes of the leaf miner, Stilbosis quadricustatella, formed after only 10 generations on its host, Quercus geminata. In contrast, some studies have found no evidence for local adaptation (Cobb and Whitham 1993;Memmott et al. (1995); Kimberling and Price 1996; Strauss 1997; Downie 1999). Memmott et al. 1995 found that the mortality of the aphid, Cinara cupressi, was similar on both novel and natal trees with equal levels of infestation.

Many researchers have also found varying degrees of local adaptation (Unruh and Luck 1987; Strauss 1990; Hanks and Denno 1993;Eliason and Potter 2000). For instance, Unruh and Luck (1987) only found differences in pinyon pine scale insect (Matsucoccus acalyptus) survival when transfers were carried out between different mountain ranges. Thus, local adaptation can occur at a range of spatial scales, from between individual hosts to between mountains. These studies suggest that populations of many insect species do not consist of one large homogeneous population, but a matrix of locally-adapted subpopulations, depending on the spatial scale at which gene flow occurs (Lajeunesse and Forbes 2002).

In New Zealand, arguably the most ecologically important herbivorous insects are the beech scale insects (Ultracoelostoma assimile and U. brittini) (Hemiptera: Margarodidae), which feed on the phloem sap of Nothofagus beech trees across approximately one million ha of the northern South Island (Beggs 2001). The large amounts of honeydew produced by beech scale insects are an important food source for many arthropods, birds, fungi and microorganisms (Hughes 1972; Gaze and Clout 1983; Clout and Gaze 1984; Didham 1993; Beggs 2001; Ewers 2002). The beech scale insect fits all the requirements of the adaptive deme formation hypothesis (see Mopper 1996; Holt and Gomulkiewicz 1997;Van Zandt and Mopper 1998) being sedentary, endophagous, and possibly facultatively parthenogenetic (Crozier 1981), since parthenogenesis is widespread within the Coccoidea (see Nur 1971; Miller and Kosztarab 1979; Gullan and Kosztarab 1997) and male beech scale insects only occur in summer but each female instar has been found in every month of the year. One of the most conspicuous features of the beech scale insect is the highly patchy distribution that it exhibits at regional (Crozier 1978) and local (Belton 1978; Gaze and Clout 1983; Kelly 1990; Didham 1993) scales. Although winged males emerge from December to March (Morales et al. 1988; Wardhaugh and Didham 2004), and first instar crawlers are present throughout the year, it is unknown how much gene flow or migration occurs between populations on different trees.

In this study the reciprocal transfers of first instar crawlers were made between red beech and mountain beech trees to ascertain if populations of beech scale insects are specializing on particular host species. Simultaneously, we also carried out reciprocal transfers of crawlers intraspecifically on red beech trees to see if demes have formed on individual trees within species.

## Materials and Methods

### Study site

This study was conducted during February and March, 2004, at the Lake Rotoiti Nature Recovery Project (41°49′ S 172°51′ E; 650 m. a. s. l.) in Nelson Lakes National Park, New Zealand. All field measurements were carried out in a 0.96 ha plot (divided into 96 10 x 10 m subplots) within a continuous beech forest, on a gentle west-facing slope over 100 m from the forest edge. The forest is dominated by red (Nothofagus fusca) and silver (N. menziesii) beech, with a few mountain (N. solandri) beech in some areas.

### Species identification

Two closely related species of Ultracoelostoma have been identified on southern beech trees in New Zealand. Identification of 30 voucher specimens of crawlers from transfer trees was undertaken using the taxonomic key in Morales (1991). However, of the three major morphological traits used by Morales (1991) to distinguish between first instar crawlers of the two species, one was indicative of the crawlers being U. assimile (there were less than 16 complex disc pores dorsally around the anus on the terminal abdominal segment) and two were indicative of the crawlers being U. brittini (the setae around the anus were pointed, rather than spatulate, and simple disc pores contained predominately four loculi). Although a definitive species identification using Morales’ (1991) descriptions was not possible, all voucher specimens did display this same mix of morphological traits, which indicates that only one species was used for transfers in this study. Detailed descriptions and illustrations of first instar U. assimile and U. brittini are given in Morales (1991) (Fig. 40 and Fig. 44, respectively) (online version available athttp://faunaseries.landcareresearch.co.nz/).

### Reciprocal transfers

First instar crawlers were collected from the lower trunks of 10 mountain beech and 20 red beech trees to use in the reciprocal transfers between host trees. Although it was impossible to be certain if crawlers were born on the trees they were collected from, a number of factors make it highly likely that it was their natal tree. First, crawlers were only collected if they were found clustered in a newly emerged group around a scale insect test on the trunk, rather than being randomly distributed. Wardhaugh and Didham (2005) showed that crawlers are retained inside the female test until all the eggs have hatched, and then emerge en masse before dispersing. Thus, the crawlers used in this study were likely to have originated from that particular host tree and to be of similar ages across all trees. Second, although beech scale insect crawlers have been shown to disperse on the wind (Morales et al. 1988; Wardhaugh and Didham 2004), members of the family Margarodidae are typically not active wind dispersers (Hanks and Denno 1998). Crawlers of actively dispersing species possess physical and behavioral adaptations to aid them in their dispersal activities (Gullan and Kosztarab 1997). The beech scale insect lacks any such obvious adaptations and appears to be highly positively thigmotactic (personal observation). Insects were incredibly reluctant to let go of the substrate they were clinging to and would even cling to tiny pieces of sooty mould fungus that would fall into the collection containers. Third, because crawlers might occasionally be blown on the wind, we took a conservative approach and only conducted transfers on calm, windless days. Therefore it is highly likely that most, if not all, of the 480 crawlers used in these experiments emerged on the trees they were collected from.

Reciprocal transfers were carried out between 10 red and 10 mountain beech trees (10 reciprocal pairs of red/mountain) to determine if scale insects have become adapted to the defensive or nutritional characteristics of their host species, or if scale insects prefer one species relative to the other. To determine if insects have become adapted to individual trees within a host species, crawlers were also reciprocally transferred between 10 red beech trees not used in the between-species transfers (five reciprocal pairs of red beech trees). Source trees used in the reciprocal transfers were selected randomly after measuring tree diameter and scale insect density at breast height (1.4 m) from every red and mountain beech tree over 5 cm in diameter at breast height within our 0.96 ha study site. The distance between each pair of randomly selected trees was recorded to determine if difference in establishment rates was related to distance from the source tree. The average distance between pairs of trees was 50.7 m (range 5 – 133 m). In each reciprocal transfer, eight crawlers from the novel host tree and eight from the natal host tree were placed in four enclosures (four natal insects in two enclosures and four novel insects in two enclosures) on each tree. Thus, 80 enclosures were used on the 20 trees in the red-mountain reciprocal transfers and 40 were used on the 10 trees in the red-red reciprocal transfers. In all cases insects were removed from, and transferred to, trunks, not branches.

**Figure 1:**
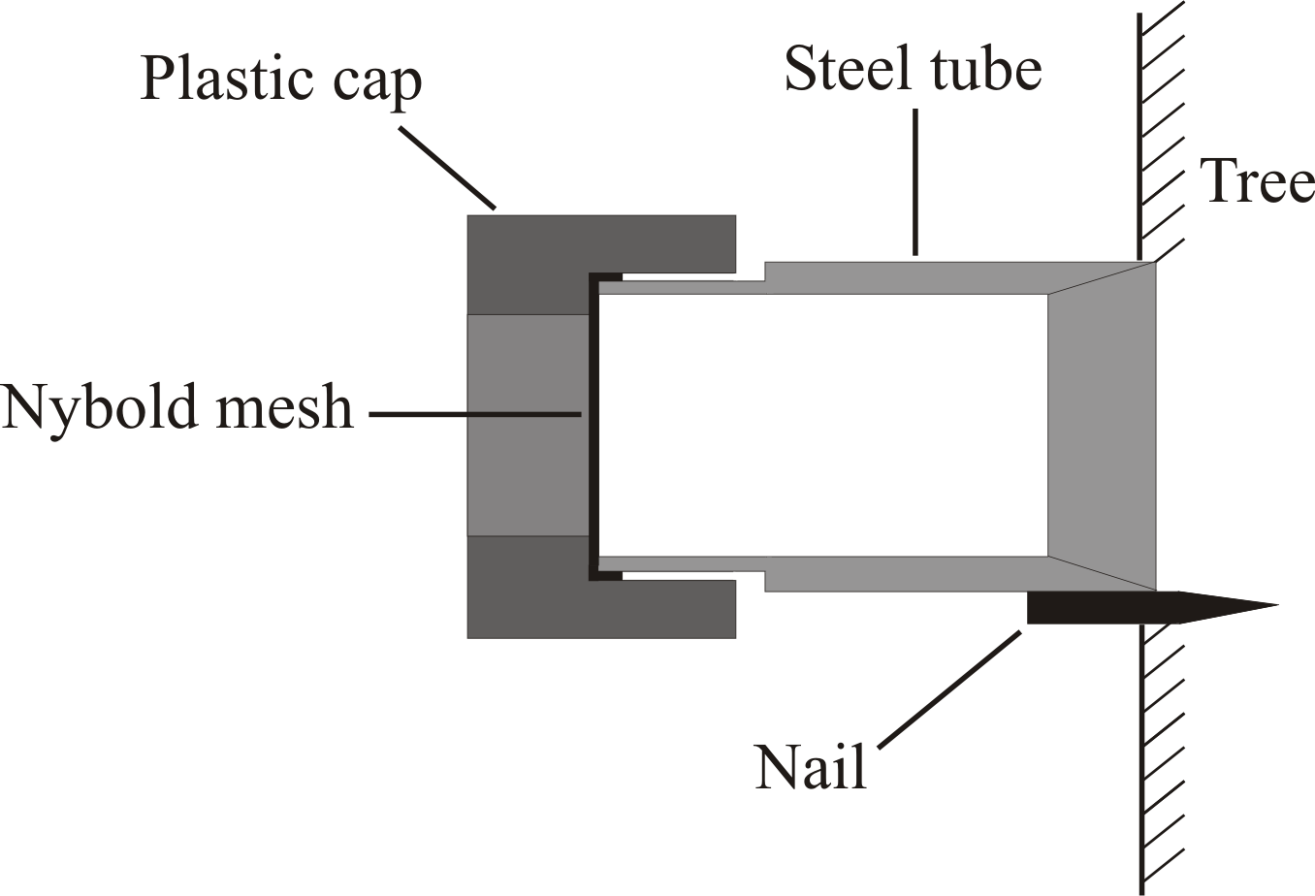
Cross section of the enclosures used for reciprocal transfers of crawlers.

Enclosures consisted of a 50 mm length of 26 mm diameter steel pipe, with a 20 mm length of 31 mm diameter plastic pipe as a removable cap (Fig. 1). To ensure that the plastic pipe fitted tightly on the end of the steel pipe, a 10 mm section of the inside of the plastic pipe was reamed out and the end 10 mm of the outside of the steel pipe was turned down in a lathe. A circular piece of nybold mesh (31 mm diameter with 250 μm mesh) was placed between the steel pipe and plastic cap to prevent crawlers from escaping out of the end. To attach the steel pipe to the tree, a headless nail was welded onto the steel pipe so that it protruded 5–8 mm past the end of the steel pipe. To prevent crawlers from escaping through small gaps between the steel pipe and the tree, the inside of the steel pipe was bored out in a lathe to create a sharp edge that pierced into the bark of the tree (but did not penetrate through to the phloem). To attach these enclosures to the tree, the steel pipe was hammered into the bark, then crawlers were placed inside and the nybold mesh was placed over the end of the steel pipe before the plastic cap was placed tightly over the end. This configuration allowed the placement and inspection of crawlers inside the enclosure without removing it from the tree.

Enclosures were placed at breast height on each tree (one per cardinal aspect) and were chosen at random to house natal or novel crawlers. It was previously determined that aspect had no effect on scale insect density at this study site (Wardhaugh et al. 2006), therefore the effect of aspect on reciprocal transfer survival rates was not explicitly tested. The area where enclosures were placed on the trunk was cleared of sooty mould and any established scale insects prior to attaching the enclosures to the tree. All crawlers, regardless of being transferred to a novel tree or being placed back on the natal tree, were held in transit for approximately the same length of time, with all transfers completed within 1 1/2 hours of collecting the crawlers. Enclosures were left for 10–14 days to allow sufficient time for crawlers to become established (McAllum 1992). A crawler was considered to be established if it had inserted its mouthparts into the bark, which was easily determined by gently nudging the insect aside to view its stylet.

The diameter at breast height (DBH) and density of scale insects on the lower trunk were recorded to determine if the size of the host tree or the local density of scale insects on the trunk influenced crawler establishment. The lower trunk density of scale insects was determined by counting the number of anal tubes protruding from the bark inside a 10 x 10 cm quadrat placed on each cardinal aspect of the tree at breast height (1.4 m).

### Statistical analyses

For the mountain to red beech reciprocal transfers (between-species), an Analysis of Covariance (ANCOVA) was performed using the arcsine square root (x + 0.25) transformed proportion of crawlers established as the dependent variable. Differences in establishment rate were tested against tree species (mountain versus red beech) and the origin of the crawlers (natal versus novel), with the distance between transfer trees, density of scale insects on the lower trunk and the DBH of the experimental trees as covariates. Because natal transfers all represent a distance of transfer of 0 m (i.e., placed back on the tree they were collected from), the covariate effect of distance on natal transfers is not meaningful. We overcame this problem by including in the analysis an interaction term between distance of transfer versus origin of the crawlers (natal versus novel).

For the red to red beech reciprocal transfers (within-species), a similar ANCOVA was performed, with the origin of the crawlers (natal versus novel) as a categorical predictor, and distance between transfer trees, tree DBH and density of scale insects on the lower trunk as covariates. All analyses were performed in Statistica version 6.0 (StatSoft 2003).

## Results

**Figure 2:**
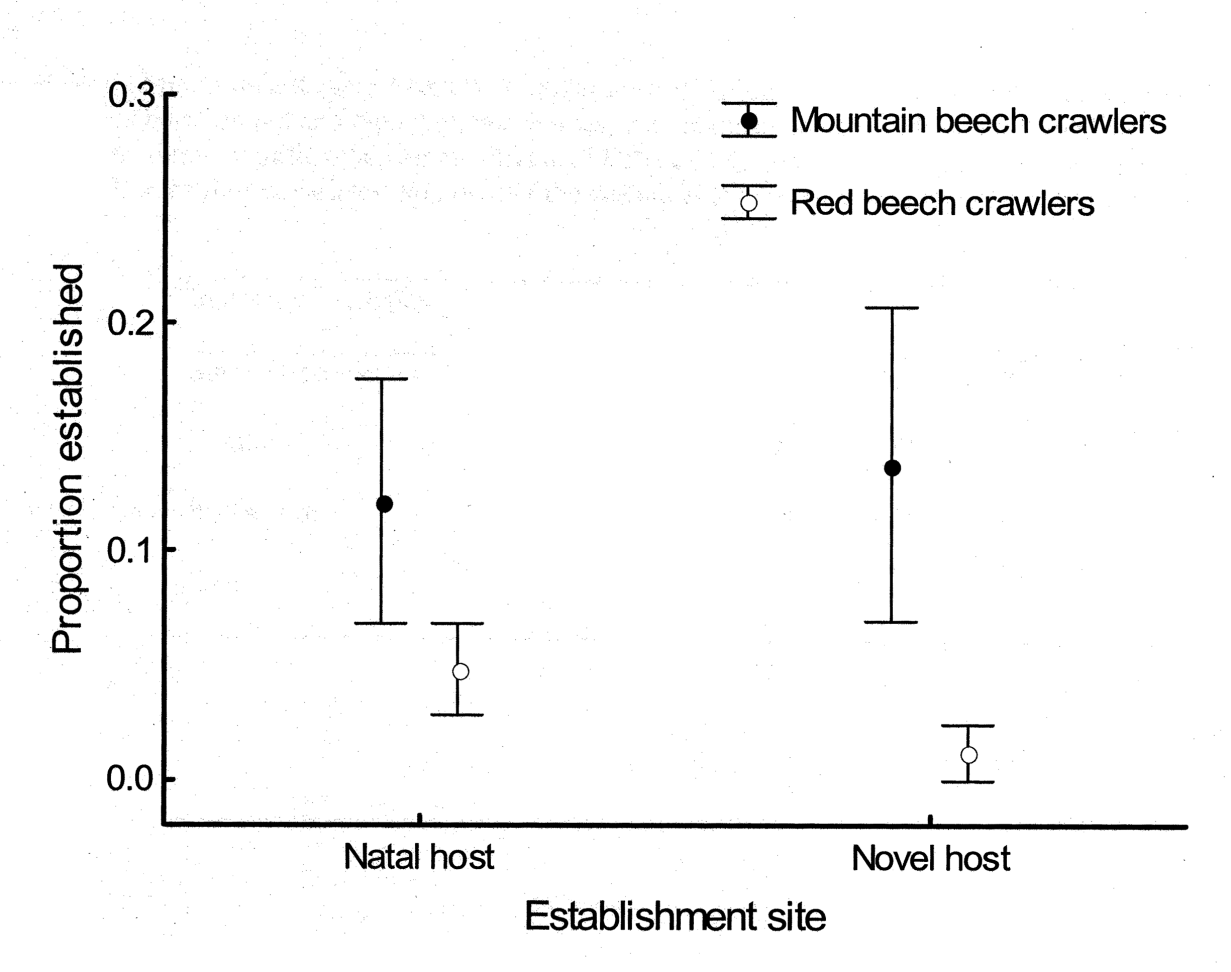
Back-transformed mean (± SE) proportion of scale insect crawlers from mountain beech (closed circles) and red beech (open circles) that successfully established on natal and novel trees.

Of the 480 crawlers used in the adaptive deme formation trials between red to red beech trees and the trials between mountain and red beeches, only 32 individuals (6.7 %) became established. In the between-species reciprocal transfers, there were no significant effects of host tree size (F _1, 32_ = 0.597, P = 0.445), distance between reciprocal pairs of trees (F _1, 32_ = 2.800, P = 0.104), or the density of scale insects on the lower trunk (F _1, 32_ = 0.797, P = 0.378) on crawler settlement, so these variables were omitted from subsequent analyses. There was no significant difference in establishment rate on novel and natal hosts in the between-species transfers (F _1, 36_ = 0.068, P = 0.796; Table 1). However, there was a significant difference in establishment rate between crawlers originating from red and mountain beech trees, with the establishment rate of crawlers from mountain beech being significantly higher than the establishment rate of crawlers from red beech (F_1, 36_ = 5.115, P = 0.030; Table 1; Fig. 2). Thus, crawlers from mountain beech trees established better on both natal mountain and novel red beech hosts, than did crawlers from red beech trees.

**Table 1:**
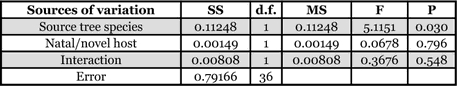
Minimum adequate ANCOVA model for establishment success of crawlers in reciprocal transfers between red and mountain beech trees, with sums of squares recalculated after the removal of non-significant covariate effects of DBH and density of scale insects on the lower trunk. Removal of covariates did not alter the statistical significance of main effects or interaction terms.

In the within-species reciprocal transfers, no evidence for adaptive deme formation within scale insect populations on red beech trees was found, and no difference was found in establishment rate between natal and novel hosts (F _1, 16_ < 0.0001, P > 0.992).

## Discussion

There was no evidence in this study that local adaptation for specific host trees or host species had occurred in Ultracoelostoma. Crawlers from mountain beech trees consistently had higher establishment rates on both natal and novel hosts than crawlers from red beech trees, indicating that scale insects from mountain beech may have higher fitness (produce more strong, healthy offspring) than those from red beech. Mountain beech crawlers even established better than red beech crawlers did on their natal red beech trees. The superior establishment rate and higher individual fitness of mountain beech crawlers may be due to a more nutritious or poorly defended host species. Thus females may molt into larger adults that produce greater numbers of better provisioned young, which can survive longer while trying to find an appropriate establishment site. The low proportion of crawlers that established in this study suggests that the number of available settlement sites may be a limiting resource. Therefore, crawlers that were longer-lived may have had more time to find the limited number of available settlement sites.

The apparent superior fitness of mountain beech crawlers, and the results from previous studies showing that mountain beech trees are often more heavily infested with scale insects than other beech tree species (Wardle 1984; but see Kelly 1990), suggests that mountain beech is the optimal host species for Ultracoelostoma. However, the specific characteristics of the host tree that affect beech scale insect fitness are unknown. One possibility is that mountain beech trees grow more vigorously than red beech trees. Scale insects are known to survive better on more vigorous, and hence more nutritious host trees (Price 1991). Rapid growth rates would also cause widespread cracking of the bark (Wardle 1984), which could increase the number of potential establishment sites. McAllum (1992) has previously shown that beech scale insect crawlers rapidly colonized newly created fissures in the bark. Indeed, our anecdotal observations suggest that establishment was higher when the experimental enclosures were situated over a crack in the bark. Similarly, Wainhouse and Howell (1983) found that transplanted crawlers of the scale insect, Cryptococcus fagisuga (Eriococcidae) on Fagus sylvatica were associated with fissures in the bark within their enclosures. However, this does not explain the higher establishment rate of mountain beech crawlers on red beech trees.

Although a number of studies have discovered preferred or more susceptible host species for herbivorous insects (e.g., Fox and Morrow 1981; Mopper et al. 1990, 1991; Preszler and Price 1995), our results are unusual in that the optimal host species was detected via the superior fitness of the focal insect rather than the superiority of the focal plant. For example, the survival rate of sawflies was significantly higher on susceptible host trees (Mopper et al. 1990), whereas in this study, the survival rate was significantly higher for crawlers from susceptible host trees. This suggests that there is a strong maternal influence on the fitness of crawlers, and that the benefits of settling on an optimal host tree are only realized via a greater growth rate and subsequent offspring fitness than via a higher establishment rate. This is an important difference since scale insect crawlers appear to be limited by the number of suitable establishment sites available.

The lack of any sympatric scale insect races on different host species suggests that either the chemical defenses of the host trees do not vary between species in ways that significantly affect scale insects, or that gene flow among trees is relatively high. Secondary chemicals, such as flavonoids, are often restricted to storage sites and may be in very dilute concentrations in the phloem (Dreyer and Jones 1981). Therefore, scale insects may bypass many of the beech tree’s chemical defenses via their feeding activities. However, the low number of crawlers that actually became established indicates that physical host tree attributes may be important to scale insect settlement. Thus, a lack of suitable establishment sites within the enclosures could have been the limited resource that restricted the settlement of crawlers.

One of the main elements of the adaptive deme formation hypothesis is that gene flow between populations of insects occupying neighboring host plants needs to be very small (Slatkin 1987). Very little is known of the reproductive behavior of the beech scale insect, therefore it is impossible to guess how much gene flow occurs between populations. Although male scale insects are relatively rare in time and space, crawlers are relatively abundant (Wardhaugh and Didham 2004), and can be blown on wind currents. Provided the immigration rate is high enough, any local selection could be swamped (Slatkin 1987; Holt and Gomulkiewicz 1997). The rate of immigration within the canopy (where most scale insects occur, Wardhaugh et al. 2006) needs to be quantified to fully assess the potential for local adaptation in the beech scale insect.

Despite the fact that no evidence for fine-scale adaptation in the beech scale insect was found in this study, it is still possible that some degree of local adaptation occurs. We only recorded the establishment rate of crawlers, so any host-driven, post-settlement factors that affect survival, growth rate or fecundity are unknown. Future studies on local adaptation in the beech scale insect should include a greater number of trees to carry out reciprocal transfers, and more crawlers should be used per transfer to control for the small proportion that become established. Microsite variation could also be controlled for (McAllum 1992) and established insects should be monitored throughout their life cycles. Furthermore, since local adaptation can occur at a range of spatial scales (Kaltz and Shykoff 1998), reciprocal transfers should be carried out between novel and natal trees separated by greater distances. Hanks and Denno (1993) only detected local adaptation for the armored scale insect when the host trees were separated by over 300 m, whereas all the host trees in this study were within 150 m of each other. Until the role of specific host tree attributes can be identified at a local scale at least, extrapolations and generalizations about the distribution and abundance of beech scale insects are likely to be inaccurate.
